# High-protein home parenteral nutrition in malnourished oncology patients: a systematic literature review

**DOI:** 10.1007/s00520-023-08218-z

**Published:** 2023-12-22

**Authors:** Paolo Cotogni, Clare Shaw, Paula Jimenez-Fonseca, Dominic Partridge, David Pritchett, Neil Webb, Amy Crompton, Pilar Garcia-Lorda, Julian Shepelev

**Affiliations:** 1 Pain Management and Palliative Care, Department of Anesthesia, Intensive Care and Emergency, Molinette Hospital and University of Turin, Turin, Italy; 2 Biomedical Research Centre at The Royal Marsden and Institute of Cancer Research, London and Sutton, UK; 3 Hospital Universitario Central de Asturias, ISPA, Oviedo, Spain; 4 Source Health Economics, Oxford, UK; 5 Open University of Catalonia, Barcelona, Spain; 6 Worldwide Medical, Health Economics and Outcomes Research, Baxter Healthcare SA, Zurich, Switzerland

**Keywords:** Parenteral nutrition, Oncology, Malnutrition, High protein

## Abstract

**Introduction:**

Up to 83% of oncology patients are affected by cancer-related malnutrition, depending on tumour location and patient age. Parenteral nutrition can be used to manage malnutrition, but there is no clear consensus as to the optimal protein dosage. The objective of this systematic literature review (SLR) was to identify studies on malnourished oncology patients receiving home parenteral nutrition (HPN) where protein or amino acid delivery was reported in g/kg bodyweight/day, and to compare outcomes between patients receiving low (< 1 g/kg bodyweight/day), standard (1–1.5 g/kg/day), and high-protein doses (> 1.5 g/kg/day).

**Methods:**

Literature searches were performed on 5^th^ October 2021 in Embase, MEDLINE, and five Cochrane Library and Centre for Reviews and Dissemination databases. Searches were complemented by hand-searching of conference proceedings, a clinical trial registry, and bibliographic reference lists of included studies and relevant SLRs/meta-analyses.

**Results:**

Nineteen publications were included; sixteen investigated standard protein, two reported low protein, and one included both, but none assessed high-protein doses. Only one randomised controlled trial (RCT) was identified; all other studies were observational studies. The only study to compare two protein doses reported significantly greater weight gain in patients receiving 1.15 g/kg/day than those receiving 0.77 g/kg/day.

**Conclusion:**

At present, there is insufficient evidence to determine the optimal protein dosage for malnourished oncology patients receiving HPN. Data from non-HPN studies and critically ill patients indicate that high-protein interventions are associated with increased overall survival and quality of life; further studies are needed to establish whether the same applies in malnourished oncology patients.

## Introduction

Malnutrition is an important problem among oncology patients, with estimated rates ranging from 30.9% to 83%, depending on cancer location and patient age [1–4]. The muscle wasting disorders cachexia and sarcopenia are commonly associated with malnutrition in cancer patients; an estimated 50–80% of cancer patients have cachexia and 20–70% have sarcopenia (depending on tumour type) [5–7]. The consequences of cancer-related muscle wasting include increased mortality [8, 9], negative effects on treatments (e.g. toxicities, termination of treatment, poor response, reduced tolerance) [8–10], and increased risk of post-operative complications [11]. Malnutrition in oncology patients can also result in decreased functional capacity [12], psychosocial symptoms [12], and lower health-related quality of life [13]. Furthermore, oncology patients who are malnourished (or who are at risk of malnutrition) spend more time in hospital [1, 14] and are readmitted more often [15, 16], constituting a substantial economic burden.

Nutritional interventions, including nutritional counselling, oral nutrition supplements, or enteral nutrition, are used to prevent or manage malnutrition. However, when these options are not feasible, are contraindicated, or ineffective, parenteral nutrition (PN) is recommended [17–20]. Parenteral nutrition is the intravenous administration of nutrients such as amino acids, glucose, lipids, electrolytes, vitamins, and trace elements, and can be delivered either at home (home parenteral nutrition [HPN]), or in a hospital setting [21].

PN is commonly used in hospitals to provide supplemental or total nutrition support to patients who are unable to maintain their nutritional status via the oral or enteral route [22]. In some cases (such as advanced oncology), patients require long-term PN, which necessitates the use of HPN [22]. However, oncology inpatients on PN (especially if intensive care unit (ICU) patients) and outpatients on HPN have completely different rates of infections as well as clinical outcomes. Whilst concerns regarding catheter-related infections had previously limited the use of HPN, its application is becoming more common, increasing by 55% in Italy between 2005 and 2012 [23].

In oncology patients, adequate protein consumption has been linked to lower rates of malnutrition, improved treatment outcomes, and longer survival [19, 24, 25]. As such, the European Society for Clinical Nutrition and Metabolism (ESPEN) guidelines recommend consuming at least 1.0 g/kg/day of protein (Table 1) [7, 19]. This recommendation is higher than the requirement for healthy individuals (0.8 g/kg/day), reflecting the positive correlation between higher protein intake, protein balance, and muscle mass [7, 19, 25]. However, increased energy and protein intake may not prevent or reduce weight loss in all patients. Anabolic resistance may be present in oncology patients, hence higher amounts of protein (≥ 1.2 and possibly up to 2 g/kg/day) may be required to balance protein synthesis than in normal individuals [7, 19, 26]. It has also been suggested that older patients with severe illness or malnutrition may need up to 2.0 g/kg/day [27]. High-protein PN (> 1.5 g/kg/day) could therefore be particularly beneficial for these patients, to rebuild muscle mass and prevent further muscle loss. High-protein PN at this dose has already been shown to be effective in other patient populations, such as critically ill patients in the ICU setting [28, 29].

**Table 1:** Overview of relevant clinical nutrition guidelines for oncology patients

Guideline	Energy	Protein
ESPEN guidelines on nutrition in cancer patients [19]	25–30 kcal/kg/day	1–1.5 g/kg/day
ESPEN guidelines on parenteral nutrition: non-surgical oncology [18]	20–25 kcal/kg/day (bedridden) or 25–30 kcal/kg/day (ambulatory patients)	1.2–2.0 g/kg/day

*ESPEN* European Society for Clinical Nutrition and Metabolism

The aim of this systematic literature review (SLR) was to understand the value of high-protein HPN and its impact on outcomes in malnourished oncology patients. Specifically, the SLR sought to identify and collate published studies on malnourished cancer patients receiving HPN, in which protein/amino acid delivery was reported in g/kg/day, to compare outcomes between patients receiving low (< 1 g/kg/day), standard (1–1.5 g/kg/day), and high-protein doses (> 1.5 g/kg/day).

## Methods

The SLR was performed in accordance with Cochrane Collaboration [30], Centre for Reviews and Dissemination (CRD) [31], and Preferred Reporting Items for Systematic Reviews and Meta-Analyses (PRISMA) guidelines [32].

### Literature sources and searches

Electronic database searches were performed on October 5^th^, 2021, in Embase, MEDLINE, the Cochrane Database of Systematic Reviews (CDSR), the Cochrane Central Register of Controlled Trials (CENTRAL), the Database of Abstracts of Reviews of Effects (DARE), the National Health Service Economic Evaluation Database (NHS EED), and the Health Technology Assessment Database (HTAD). All databases were searched via the Ovid platform, with the searches date-limited from 2005 to ensure that only contemporary data were captured. The database searches were complemented by hand-searching of the National Institutes of Health (NIH) trial registry (https://clinicaltrials.gov/), and the proceedings of seven oncology- and nutrition-themed conferences held since January 2019. Conference hand-searching was date-limited from 2019 onwards as it was presumed that any high-quality abstracts presented before this date would now be available as full publications. The bibliographic reference lists of included studies and relevant SLRs and meta-analyses identified during screening were also hand-searched. Full details of the SLR search strategy are provided in Supplement 1. The SLR protocol was not pre-registered in any protocol registry or online repository.

### Study selection criteria

The population, intervention, comparator(s), outcomes, and study design (PICOS) elements used to assess study eligibility are presented in Supplement 2. Studies were eligible for inclusion if they reported on malnourished oncology outpatients receiving HPN, and protein or amino acid delivery was reported in g/kg/day. Oncology patients were considered to be malnourished if: (a) the publication explicitly described patients as having malnutrition of any kind, low body weight, clinically significant weight loss, low body mass index (BMI), clinically significant BMI reduction, cachexia, sarcopenia, or muscle wasting/loss/atrophy; and/or (b) cancer stage was described as incurable, non-curative, palliative, end-of-life, advanced, metastatic, late stage, stage IV, or hospice-treated. This approach was adopted given that patients with advanced cancer are typically only prescribed PN if malnourished or have a non-functioning gastrointestinal tract [19]. Eligible study designs included randomised controlled trials (RCTs), non-randomised multi-arm trials, single-arm trials, and prospective or retrospective observational studies. Eligible studies must have been written in English, although there were no restrictions on country of origin.

### Screening and extraction

All publications were screened against the predefined eligibility criteria by two independent reviewers at both the title/abstract and full-text screening stages. Any conflicts were resolved via dialogue between the two reviewers, and where necessary, a third reviewer provided arbitration. Full lists of included and excluded publications are provided in Supplement 3. Data from included publications were extracted into standardised data extraction tables in Microsoft® Excel by one individual, with all information checked and validated by a second individual. Data extracted from eligible publications included the country of origin, study design, study dates, sample size, participant age, sex, cancer stage and location, performance status, nutritional status, PN type (total or supplemental), protein dose, and key study findings, including clinical, safety, and quality of life outcomes. Energy data provided in kJ were converted into kcal by dividing by 4.184. No formal risk of bias assessment was performed due to the heterogeneity of study designs encountered, and the fact that quantitative data synthesis was not conducted.

## Results

**Fig. 1:**
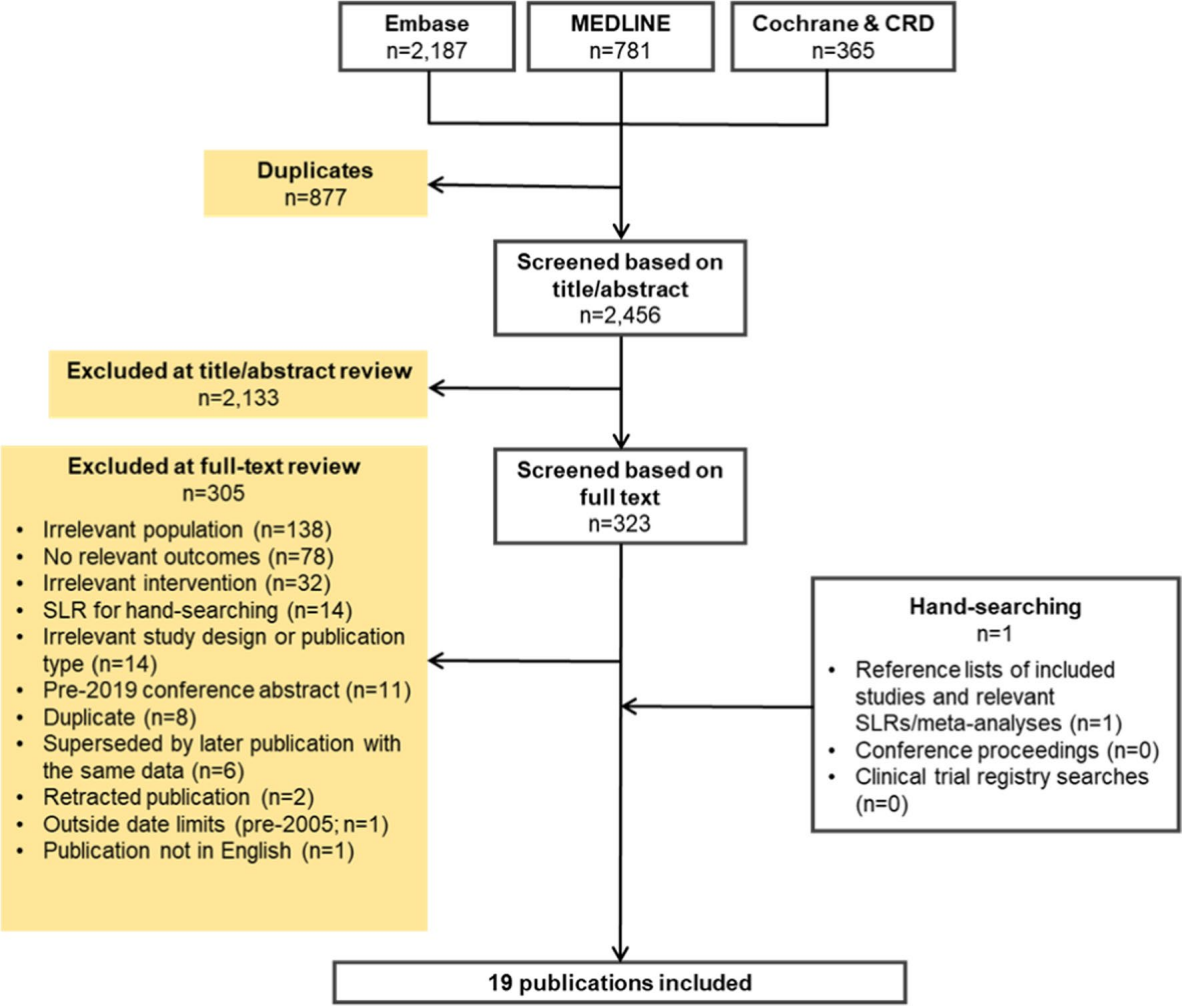
PRISMA diagram. Abbreviations: CRD, Centre for Reviews and Dissemination; PRISMA, Preferred Reporting Items for Systematic literature reviews and Meta-analyses; SLR, systematic literature review

The electronic database searches identified 3,333 citations. After removal of 877 duplicates, 2,133 publications at the title/abstract screening stage, and 305 publications at the full-text screening stage, 18 publications from the electronic database searches were deemed eligible for inclusion in the SLR. Hand-searching yielded one additional eligible publication, resulting in a total of 19 publications included in the SLR (Fig. 1).

### Study and patient characteristics

Detailed study characteristics are presented in Table 2. The 19 included publications consisted of one RCT [33], one single-arm trial [34], 10 prospective observational studies [35–44], and seven retrospective observational studies [24, 45–50]. The most common country of origin was Italy (11 publications) [35, 36, 38–42, 46–48, 50], followed by Denmark (four publications) [33, 35, 36, 45]. Sample size in the included publications ranged from 19 [50] to 1,014 [47], while the age of patients was between 48.8 (mean) [49] and 68 years (median) [41, 43]. The most common types of cancer were gastrointestinal (17 publications) [24, 33, 35–39, 41–50], pancreatic (12 publications) [24, 33–36, 38, 39, 41, 44, 47, 48, 50], and ovarian (11 publications) [35, 36, 38, 40–42, 44, 47–50]. Cancer stage was described as advanced or Stage III in 10 publications [24, 38–44, 47, 49], metastatic or Stage IV in nine publications [34, 37–43, 45], and incurable, palliative, or terminal in nine publications [24, 33, 35, 36, 45–48, 50]. Twelve publications reported details of prior or concurrent anticancer treatments [24, 33, 35, 37–44, 47, 49], while seven publications did not [24, 34, 36, 45, 46, 48, 50].

**Table 2:** Characteristics of studies identified by the SLR

Publication Authors (year) Study period, Country Setting	N	Age	Females, *n* (%)	Cancer location, *n* (%)	Tumour stage, *n* (%)	Treatment	Baseline PS	Baseline NS
Randomised controlled trials								
Obling et al. (2019) [33] May 2014 to Nov 2016 Denmark	47 (22PN vs 25 control)	Median (range) age in years: All patients: 66.9 (41.5–88.2) Non-sHPN group: 65.9 (43.3–88.2) sHPN group: 67.4 (41.5–81.6)	All patients: 17 (36) Non-sHPN group: 10 (40) sHPN group: 7 (32)	Oesophagus: 2 (4) Stomach: 9 (19) Duodenum: 2 (4) Pancreas: 27 (58) Bile duct: 2 (4) Colorectal: 5 (11)	Incurable (locally advanced or metastatic): 47 (100)	91% received palliative chemotherapy at study inclusion	Unspecified PS scale, all patients, *n* (%): 0: 6 (13) 1: 25 (53) 2: 16 (34) non-sHPN group, *n* (%): 0: 5 (20) 1: 13 (52) 2: 7 (28) sHPN group, *n* (%): 0: 1 (5) 1: 12 (54) 2: 9 (41)	Median (range) BMI: All patients: 21.3 (14.8–35.7) non-sHPN group: 21.3 (15.9–29.6) sHPN group (*n* = 22): 21.6 (14.8–35.7) WL prior to inclusion, *n* (%): < 5%: 8 (17) 5–10%: 10 (22) > 10%: 29 (61)
Prospective observational studies								
Bozzetti et al. (2014) [35] Nov 2004 to March 2011 Italy, France, Germany, Poland, Netherlands, Spain, Belgium, Denmark, UK, Canada	414	Median (range): 62 years (16–90 years)	190 (45.9)	Head and neck: 50 (12.1) Stomach: 92 (22.2) Small bowel-biliary: 10 (2.4) Colon-rectum: 84 (20.3) Ovary: 51 (12.3) Pancreas: 46 (11.1) Other: 81 (19.6)	Incurable: 414 (100) Locoregional: 131 (32.2) Metastatic: 105 (25.8) Locoregional and metastatic: 171 (42.0) Not applicable: 8 (NR)	Most recent therapy, *n* (%): Surgery: 53 (16.9) Radiotherapy: 24 (7.6) Chemotherapy, 1st line: 89 (28.3) Chemotherapy, 2nd line: 71 (22.6) Chemotherapy, 3rd line: 77 (24.5) NA: 100 (NR)	Median (range) KPS: 60 (20–100) (26 patients with missing data)	WL (habitual weight), median (range): 24% (–8 to –56); WL (previous 6 months), median (range): 16% (–44 to –‍50); BMI, median (range): 19.5 (12.8–30.0)
Bozzetti et al. (2015) [36] Nov 2004 to March 2011 Italy, France, Germany, Poland, Netherlands, Spain, Belgium, Denmark, UK, Canada	579	Median (range): 64 years (16–95 years)	262 (47.3)	Colon-rectum: 108 (18.7) Head and neck: 67 (11.6) Ovary: 64 (11.1) Pancreas: 76 (13.1) Small bowel-biliary: 13 (2.2) Stomach: 136 (23.5) Other: 115 (19.9)	Incurable: 579 (100) Metastatic: 379 (66.3) Non-metastatic: 193 (33.7) Data missing on presence of metastases: 7 (NR)	NR	Median (range) KPS: 60 (20–100) (26 patients with missing data)	WL (previous 6 months), median (range): 13.8% (–44 to –50); BMI, median (range): 20.0 (12.7–34.2)
Culine et al. (2014) [37] Sept 2009 to March 2010 France	749	Mean (SD): 63.0 years (11.4)	296 (39.5)	Digestive system: 370 (49.9) Oropharyngeal: 148 (20.0) Respiratory system: 109 (14.7) Other sites: 132 (17.8)	Metastatic: 489 (65.3)	Ongoing chemotherapy or/and radiotherapy: 702 (98.3%)	Unspecified performance status scale, *n* (%): 0–1: 210 (28.2) 2: 354 (47.5) 3–4: 181 (24.3)	Mean body mass (SD): 59.2 (12.3); mean (SD) WL in last 6 months: 15.6% (7.7%); mean (range) BMI: 21.2 (12.7–39.1)
Cotogni et al. (2017) [38] Oct 2011 to Sept 2013 Italy	111	Median (range) age in years: 62 (32–79)	54 (49)	Stomach: 38 (34) Colon/rectum: 21 (19) Pancreas/biliary system: 20 (18) Oesophagus: 10 (9) Lung: 10 (9) Ovary: 2 (2) Others: 10 (9)	III: 25 (23) IV: 86 (77) Metastatic: 73 (68)	Patients receiving chemotherapy and/or radiation therapy: 72 (65%) Patients receiving no concurrent treatment: 39 (35%)	KPS median (range): 70 (60–80)	Median (range) BMI: 20.7 (13.5–29.5); median (range) WL in 3 months before HPN: 11.7% (0–38.3)
Cotogni et al. (2018) [39] Oct 2014 to March 2015 Italy	65	Median (range) age in years: 64 (33–79)	32 (49)	Stomach: 26 (40) Pancreas/biliary system: 15 (23) Colon/rectum: 9 (14) Gynaecological: 5 (8) Oesophagus: 3 (4) Other: 7 (11)	III: 16 (25) IV: 49 (75) Metastatic: 46 (71)	Patients were receiving: Chemotherapy: 65/65 (100%) Neoadjuvant chemotherapy: 9/65 Adjuvant chemotherapy: 37/65 Palliative chemotherapy: 19/65 Chemotherapy and radiation therapy together: 10/65 Adjuvant chemotherapy following surgery: 37/65 Neoadjuvant chemotherapy and subsequently underwent surgery: 5/65 Patients that received surgery: 42 (65%)	KPS mean (range): 67.4 (50–90), median: 70.0	Mean (range) body weight in kg: 57.4 (33.0–100.0), median: 57.0; median (range) WL in 3 months: 10.7 (1.9–27.8); Mean (range) BMI: 21.2 (12.7–39.1), Median: 21.1
Cotogni et al. (2020) [40] June 2008 to May 2015 Italy	761	Mean (SD): 62.90 (10.28)	380 (49.9)	Ovary: 47 (6.2) Gastrointestinal: 564 (74.1) Other: 150 (19.7)	II/III: 220 (28.9) IV: 541 (71.1)	Patients receiving chemotherapy, *n* (%) SPN and chemotherapy: 376 (49.4) TPN and chemotherapy: 99 (13.0) Patients not receiving chemotherapy, *n* (%) SPN and no chemotherapy: 191 (25.1) TPN and no chemotherapy: 95 (12.5)	KPS, *n* (%): 50: 102 (13.4) 60: 222 (29.2) 70: 387 (50.9) 80–90: 50 (6.6)	Mean body mass (SD): 58.42 kg (10.92), mean BMI (SD): 21.37 (3.76), WL in 3 months before HPN, *n* (%): ≤ 10.0%: 233 (30.6); 10.1–15%: 187 (24.6); 15.1–20%: 181 (23.8); > 20%: 160 (21.0)
Cotogni et al. (2021) [42] June 2008 to May 2015 Italy	761	Median (IQR) age in years: 64 (57–70)	380 (50)	Gastrointestinal: 564 (74) Ovary: 47 (6) Others: 150 (20)	II/III: 220 (29) IV: 541 (71)	475 patients (62%) received anticancer treatments (CT/ RT)	KPS, *n* (%): 50: 102 (13) 60: 222 (29) 70: 387 (51) 80–90: 50 (7)	Median (IQR) body mass in kg: 57.3 (50.4–65) Median (IQR) BMI: 21 (18.7–23.4) Median (IQR) % WL in 3 months before HPN: 13.8 (9.3–19.1)
Cotogni et al. (2022) [41] Oct 2012 to Sept 2015 Italy	215	Median (IQR) age in years: HPN group (*n* = 89): 68 (62 − 74) non-HPN group (*n* = 36): 68 (63–74)	HPN group: 42 (47) non-HPN group: 15 (42)	HPN group: Oesophagus/stomach: 25 (28) Pancreas/biliary system: 21 (24) Colon/rectum: 13 (15) Ovary: 7 (8) Lung: 4 (4) Urinary tract/prostate: 3 (3) Other: 16 (18) Non-HPN group: Oesophagus/stomach: 8 (22) Pancreas/biliary system: 9 (25) Colon/rectum: 2 (6) Ovary: 2 (6) Lung: 3 (8) Urinary tract/prostate: 3 (8) Other: 9 (25)	HPN group (*n* = 89): II: 3 (3) III: 13 (15) IV: 73 (82) Non-HPN group (*n* = 36): II: 1 (3) III: 7 (19) IV: 28 (78)	Patients receiving chemotherapy, *n* (%): HPN group (*n* = 89): 0 (0) non-HPN group (*n* = 36): 0 (0)	Median (IQR) KPS: HPN group: 60 (50 − 70) non-HPN group: 60 (60 − 70) ECOG PS: HPN group, *n* (%) 1: 1 (1) 2: 60 (67) 3: 28 (32) non-HPN group, *n* (%) 1: 0 (0) 2: 29 (81) 3: 7 (19)	Median (IQR) body weight in kg: HPN group: 57 (50–65) non-HPN group: 58 (51–69) Median (IQR) %WL in 3 months before HPN: HPN group: 10.9 (7.0 − 16.7) non-HPN group: 11.3 (6.5 − 16.0)
Ma et al. (2021) [43] Oct 2014 to Jan 2019 Taiwan	50	Median (range) age in years in PN group: 68 (35–88)	11 (22)	Gastric: 25 (100)	Locally advanced: 10 (40) Metastatic: 15 (60)	All patients were undergoing salvage CT	ECOG PS in PN group, *n* (%) 1: 11 (44) 2: 14 (56)	Body mass mean (kg) PN group: 51.66 (10.53) BMI mean PN group: 20.1 (3.6)
Vashi et al. (2014) [44] April 2009 to March 2014 USA	52	Mean 53.2 (9.4)	31 (59.6)	Pancreas: 14 (26.9) Colorectal: 11 (21.2) Ovarian: 6 (11.5) Appendix: 5 (9.6) Stomach: 4 (7.7) Others: 12 (23.1)	Advanced: 52 (100)	All patients underwent active CT, RT, or hormonal therapy while enrolled in the study	Mean (SD) KPS: 60.1 (10.8)	Body mass mean (SD) kg: 62.2 (14.6) Mean (SD) % WL in 6 months prior to HPN: 16.9 (9.3) Mean WL in 6 months prior to HPN, in kg: 13.2
Single arm trial								
Pelzer et al. (2010) [34] Jan 2002 to Jan 2004 Germany	32	Assumed to be median, (range) in years: 62 (47–75)	14 (43.8)	Pancreas: 32 (100)	IV inoperable: 32 (10	NR	NR	Median (range) BMI: 19.7 (14.4–25.9)
Retrospective observational studies								
Goodrose-Flores et al. (2020) [24] 2016 to 2018 Sweden	Total: 124 Standard PN: 104 High-protein PN: 20	All patients: 63.4 (12.9) Standard PN 63.4 (13.1) High-protein PN 63.4 (12.3)	All patients: 60 (48) Standard PN: 50 (48) High-protein PN: 10 (50)	Standard PN group Gastrointestinal: 41 (39) Pancreatic: 17 (16) Gynaecological: 12 (12) Head–neck: 6 (6) Other: 28 (27) High-protein PN group (*n* = 20) Gastrointestinal: 9 (45) Pancreatic: 3 (15) Gynaecological: 1 (5) Head–neck: 2 (10) Other: 5 (25)	Advanced/palliative: 124 (100)	Treatment with curative intent: 0	NR	Standard PN: 65.2 (14.1) High-protein PN: 63.2 (10.1) All patients: 22.7 (4.3) Standard PN: 22.8 (4.6) High-protein PN: 21.9 (3.0)
Obling et al. (2018) [45] Jan 2005 to Dec 2015 Denmark	80	Median (CI) age: 63.8 (25.1, 83.6)	53 (66)	Upper GI cancer: 40 (50) Lower GI cancer: 10 (12) Gynaecological cancer: 22 (28) Other cancer: 8 (10)	Incurable: 80 (100) Metastatic: 71 (89)	NR	NR	Median (range) BMI: All patients: 20.7 (14.1–38.5)
Ruggeri et al. (2020) [46] July 1990 to July 2019 Italy	969	Mean PN group: 64.2 (12.6)	PN group: 305 (48.5)	PN group: GI tract: 319 (62.2) Head-neck: 104 (16.5) Other organs: 114 (18.1) Lung: 20 (3.2)	PN group: Palliative: 666 (100)	NR	KPS in PN group, *n* (%): 40: 157 (25.0) 50: 276 (43.9) 60: 127 (20.2) 70: 53 (8.4) 80: 16 (2.5)	NR
Ruggeri et al. (2021) [47] July 1990 to June 2020 Italy	1,014	Mean PN group: 64.6 (12.4)	PN group: 222 (49.2)	PN group: Head and neck: 116 (17.4) Oesophagus: 47 (7) Stomach: 153 (23) Pancreas: 65 (9.8) Colon: 128 (19.2) Lung: 21 (3.1) Breast: 16 (2.4) Uterus: 14 (2.1) Ovary: 59 (8.9) Other organs: 47 (7.1)	Advanced, palliative cancer patients in PN group: 629 (100)	Treatment, *n* (%): CT: 14 (36.8) CT-RT: 1 (2.6) None: 23 (60.5)	Mean (SD) KPS: 62.7 (18.53)	NR
Santarpia et al. (2006) [48] Jan 1996 to Sept 2003 Italy	152	Mean (SD): 57.8 (13.6)	107 (70.4)	Stomach: 48 (NR) Ovaries: 42 (NR) Colorectum: 30 (NR) Endometrium: 7 (NR) Breast: 6 (NR) Ileum: 5 (NR) Gallbladder: 4 (NR) Pancreas: 3 (NR) Kidney: 2 (NR) Skin: 1 (NR) Prostate: 1 (NR) Abdominal sarcoma: 1 (NR) Unknown: 2 (NR)	Terminal: 152 (100)	NR	KPS ≤ 40: 51.3% KPS ≥ 50: 62 patients	Body mass (kg) (SD): 53.4 (10.9) BMI mean (SD): 20.1 (3.6) Mean (SD) WL in previous 6 months, in kg: 9.5 (4.7)
Soo et al. (2008) [49] 1999 to 2006 Canada	38	Mean SD: 48.76 (13.8)	27 (71)	Ovarian: 13 (34) Colonic: 6 (15.8) Gastric: 6 (15.8) Peritoneal: 3 (7.9) Unknown: 2 (5.3) Oesophageal: 2 (5.3) Carcinoid: 1 (2.6) Cervical: 1 (2.6) Ampullary: 1 (2.6) GIST: 1 (2.6) Anaplastic large-cell lymphoma: 1 (2.6) Rectal: 1 (2.6)	Advanced: 38 (100)	Treatment, *n* (%): CT: 14 (36.8) CT-RT: 1 (2.6) None: 23 (60.5)	Mean (SD) KPS: 62.7 (18.53)	NR
Santarpia et al. (2018) [50] Dates NR Italy	19	Mean: 59 (9.0)	10 (52.6)	Ovary: 8 (NR) Stomach: 4 (NR) Colon-rectum: 3 (NR) Pancreas: 2 (NR) Gallbladder: 1 (NR)	Late-stage incurable: 19 (100)	NR	NR	Mean body mass: 50.5 (8.6) WL in the previous 6 months: in kg: 11.8 ± 4.5 %: 23.5 ± 8.1

*BMI* body mass index, *CI* confidence interval, *CT* chemotherapy, *ECOG* Eastern Cooperative Oncology Group, *GI* gastrointestinal, *GIST* gastrointestinal stromal tumour, *IQR* interquartile range, *KPS* Karnofsky Performance Status, *NR* not reported, *NS* nutritional status, *PN* parenteral nutrition, *PS* performance status, *RT* radiotherapy, *SD* standard deviation, *sHPN* supplemental home parenteral nutrition, *SLR* systematic literature review, *SPN* supplemental parenteral nutrition, *TPN* total parenteral nutrition, UK, United Kingdom; USA, United States of America, *WL* weight loss

### Overview of PN intervention reported

Details of the PN interventions are described in Table 3. The type of PN was a mix of total and supplemental PN in seven publications [37, 39–42, 44, 47], total PN alone in five publications [35, 36, 48–50], supplemental PN alone in five publications [33, 34, 38, 43, 45], and was unclear in the two remaining publications [24, 46]. Protein dose ranged from 0.77 to 1.5 g/kg/day [24]. Sixteen publications investigated standard-protein doses (1–1.5 g/kg/day) [33–42, 44, 46–50], two reported on low-protein doses (< 1 g/kg/day) [43, 45], and one included both [24], but none involved high-protein doses (> 1.5 g/kg/day). For publications that reported a target dose but not the dose delivered, it was assumed that the target dose was the dose delivered. In two publications [35, 36], targeted protein delivery was reported only as ≥ 1 g/kg/day; in the absence of an upper limit, it was assumed that targeted protein delivery fell within the standard range (1–1.5 g/kg/day). Seventeen publications reported energy intake [33, 35–50], which ranged between 19.7 [24] and 40.2 [33] kcal/kg/day. The duration of PN administration was reported in nine studies [24, 33–35, 38, 42–45]; the shortest duration was 28 days (total) [37] and the longest was 364.9 days (median) [45].

**Table 3:** Parenteral nutrition intervention details

Publication Authors (year)	PN intervention		Change in body mass
	Type of PN and duration of PN	Dose planned; dose administered	
Randomised controlled trials			
Obling et al. (2019) [33]	Type: Supplemental Duration: 17/47 (36%) patients completed 24 weeks of treatment	Dose planned: Energy: 30 kcal/kg/day; protein: 1.5 g/kg/day Dose given: Median energy intake in non-sHPN group: 31.1–40.4 kcal/kg/day, median energy intake in sHPN group: 30.0–40.2 kcal/kg/day Median protein intake in non-sHPN group: 1.10–1.16 g/kg/day Median protein intake in sHPN group: 1.08–1.39 g/kg/day	Median (range) BMI in the non-sHPN group: - Baseline: 21.3 (15.9–29.6) - Visit 2: 21.0 (14.4–29.8) - Visit 3: 19.5 (15.9–29.8) - Visit 4: 22.9 (15.8–31.7) - Visit 5: 22.9 (15.6–31.6) Median (range) BMI in the sHPN group: - Baseline: 21.5 (14.8–35.7) - Visit 2: 22.2 (16.1–36.8) - Visit 3: 22.7 (16.5–38.8) - Visit 4: 23.7 (16.9–34.8) - Visit 5: 23.5 (18.9–31.6
Prospective observational studies			
Bozzetti et al. (2014) [35]	Type: Total Duration: Median (range): 2 months (1–126) (*n* = 139)	Dose planned: Energy: at least 25 kcal/kg/day, protein: 1 g amino acid/kg/day Dose given: NR	NR
Bozzetti et al. (2015) [36]	Type: Total Duration: NR	Dose planned: Energy: at least 25 kcal/kg/d, protein: 1 g amino acid/kg/day Dose given: NR	NR
Culine et al. (2014) [37]	Type: Total and supplemental Duration: NR	Dose planned: NR Dose given: Energy, mean (SD): 26 (0.4) kcal/kg/day; protein, mean (SD): 1.15 (0.02) g/kg/day	Weight in kg, mean (SD): - Baseline: 59.6 (12.5) - Day 28: 61.1 (12.5) - p < 0.001 BMI, mean (SD): - Baseline: 20.9 (4.0) - Day 28: 21.4 (3.9) - p < 0.001
Cotogni et al. (2017) [38]	Type: Supplemental Duration: Median (range) duration of PN in days: 137 (21–576)	Dose planned: Energy for bedridden patients: 20–25 kcal/kg/day, energy for ambulatory patients: 25–30 kcal/kg/day, protein: 1–1.5 g/kg/day Dose given: NR	NR
Cotogni et al. (2018) [39]	Type: Total and supplemental Duration: NR	Dose planned: Energy: 25–30 kcal/kg/day, protein: 1–1.5 g/kg/day Dose given: NR	Mean (range), median - Baseline: 57.4 (33.0–100.0), 57.0 - 60 days: 58.9 (37.7–96.0), 57.0 - 90 days: 59.7 (37.5–95.0), 60.0 - p < 0.01
Cotogni et al. (2020) [40]	Type: Total and supplemental Duration: NR	Dose planned: Energy: 25–30 kcal/kg/day, protein: 1–1.5 g/kg/day Dose given: NR	NR
Cotogni et al. (2021) [42]	Type: Total and supplemental Duration: Median (IQR) duration in days: 203 (101–296)	Dose planned: Energy: 25–30 kcal/kg/day, protein: 1–1.5 g/kg/day Dose given: NR	NR
Cotogni et al. (2022) [41]	Type: Total and supplemental Duration: NR	Dose planned: Energy: 25–30 kcal/kg/day, protein: 1–1.5 g/kg/day Dose given: NR	NR
Ma et al. (2021) [43]	Type: Supplemental Duration: Median (range) duration in days: 135 (16–569)	Dose planned: Energy: 910–1,800 kcal/day; protein: 33–60 g/day Dose given: NR	Mean (SD) body weight in PN group, in kg: - Baseline (*n* = 25): 51.66 (10.53) - Change from baseline at 0.5 months (*n* = 22): –1.14 (4.14), *p* = 0.212 - Change from baseline at 1 month (*n* = 19): 4.13 (25.54), *p* = 0.490 - Change from baseline at 2 months (*n* = 12): 1.93 (5.24), *p* = 0.227 - Change from baseline at 3 months (*n* = 7): 3.34 (4.72), *p* = 0.110 Mean (SD) BMI in PN group: - Baseline (*n* = 25): 20.1 (3.6) - Change from baseline at 0.5 months (*n* = 22): –0.4 (1.7), *p* = 0.285 - Change from baseline at 1 month (*n* = 19): –0.4 (2.0), *p* = 0.422 - Change from baseline at 2 months (*n* = 12): 0.9 (2.4), *p* = 0.248 - Change from baseline at 3 months (*n* = 7): 1.3 (1.8), *p* = 0.105
Vashi et al. (2014) [44]	Type: Total and supplemental Duration: Mean (SD) (range) duration in months: 3.4 (2.5) (0.4–11.7)	Dose planned: Energy: 25–30 kcal/kg for BMI < 30; 22–25 kcal/kg for BMI ≥ 30; protein: 1.5–2 for BMI < 30; 2–2.5 for BMI ≥ 30 Dose given: Mean (SD) PN energy delivery in patients with < 3 months follow-up (*n* = 37) in kcal/kg/day: 23.4 (5.2); Mean (SD) PN energy delivery in patients with ≥ 3 months follow-up (*n* = 15) in kcal//day: 20.8 (3.9); protein < 3 months follow-up 1.5 g/kg/day & ≥ 3 months follow-up 1.3 g/kg/day	Mean (SD) body weight in kg amongst patients with 1 month of follow-up data (*n* = 39): - Baseline: 61.5 (13.4) - Month 1: 63.1 (12.7) - *p* = 0.03
Single arm trial			
Pelzer et al. (2010) [34]	Type: Supplemental Duration: Median (range) duration in weeks: 18 (8–35)	Dose planned: Protein: 1.2–1.5 g/kg/day Dose given: NR	Median (range) BMI: - Before PN: 19.7 (14.4–25.9) - After PN: 20.5 (15.4–25.0)
Retrospective observational studies			
Goodrose-Flores et al. (2020) [24]	Type: Unclear Duration: Median duration of PN across all patients: 2 months	Dose planned: NR Dose given: Mean (SD) energy intake in kcal g/kg/day: • Standard PN (*n* = 104): 19.7 (4.7), High-protein PN (*n* = 20): 21.4 (5.1) Protein: Mean (SD) protein intake in g/kg/day: • Standard PN (*n* = 104): 0.77 (0.19) • High-protein PN (*n* = 20): 1.15 (0.26)	Mean (SD) body weight in kg in standard PN group (*n* = 104): - Before PN: 65.2 (14.1) - After PN: 65.5 (13.1) - % change from baseline: 0.12 (5.3) Mean (SD) BMI in standard PN group (*n* = 104): - Before PN: 22.8 (4.6) - After PN: 22.9 (4.4)
Obling et al. (2018) [45]	Type: Supplemental Duration: Median (range) duration in days: all patients (*n* = 80): 364.9 (6–2,006)	Dose planned: Energy: 30 kcal/kg/day, protein: 1.2 g/kg/day Dose given: Median (range) actual protein intake: all patients: 0.87 (0.43–1.92)	NR
Ruggeri et al. (2020) [46]	Type: Unclear Duration: NR	Dose planned: NR Dose given: Energy, mean (SD): 32 (8) kcal/kg/day, protein, mean (SD): 1.4 (0.4) g/kg/day	NR
Ruggeri et al. (2021) [47]	Type: Total and supplemental Duration: NR	Dose planned: NR Dose given: Energy, mean (SD): 31 (9) kcal/day, protein, mean (SD): 1.3 (0.4)	Mean (SD) change in BMI after 1 month of treatment: - TPN: 0.04 (0.25) - SPN: 0.21 (0.23)
Santarpia et al. (2006) [48]	Type: Total Duration: NR	Dose planned: Energy: 20–30 kcal/kg/day, protein: 1–1.5 g/kg/day Dose given: NR	Mean (SD) weight in kg amongst the 64 patients that survived longer than 60 days: - Baseline: 51.7 (10.3) - After 1 month: 53.2 (10.3) - *p* = 0.0001 Mean (SD) BMI amongst the 64 patients that survived longer than 60 days: - Baseline: 19.6 (3.1) - After 1 month: 20.1 (3.1) - *p* = 0.0001
Soo et al. (2008) [49]	Type: Total Duration: NR	Dose planned: Energy: 25 kcal/kg, protein: 1 g/kg/day Dose given: NR	NR
Santarpia et al. (2018) [50]	Type: Total Duration: NR	Dose planned: NR Dose given: Energy: 30 kcal/kg/day, protein: 1.34 g/kg/day	NR

*BMI* body mass index, *IQR* interquartile range, *NR* not reported, *PN* parenteral nutrition, *SD* standard deviation, *sHPN* supplemental home parenteral nutrition

### Change in body mass

Nine studies reported changes in body mass or BMI (Table 3) [33, 34, 37, 39, 43–45, 47, 48]. Of these, Culine et al. (2014) [37], Cotogni et al. (2018) [39], Vashi et al. (2014) [44], Goodrose-Flores et al. (2020) [24], and Santarpia et al. (2006) [48] reported that receipt of protein-containing HPN significantly increased body mass or BMI over the course of the study period, although all studies administered standard doses of protein (range: 1–1.5 g/kg).

The RCT by Obling et al. (2019) [33] reported that, with standard protein doses, median BMI increased between baseline and visit 5 in both the non-supplemental HPN (sHPN) group (best practice nutritional care and dietetic counselling) (from 21.3 to 22.9 m/kg^2^) and the sHPN group (sHPN and dietetic counselling) (from 21.5 to 23.5 m/kg^2^), but did not report whether the change from baseline was significant. Ruggeri et al. (2021) [47] reported that one month of sHPN increased BMI to a greater extent than one month of total HPN (0.21 vs 0.04, respectively), with a mean protein dose of 1.3 g/kg, but did not report whether the change from baseline was significant.

Two studies, Ma et al. (2021) [43] and Pelzer et al. (2010) [34], reported that low to standard protein (0.6–1.5 g/kg/day) did not significantly increase body weight or BMI during the study period.

### Change in other outcomes

Due to the high heterogeneity in the other outcomes examined, only change in body mass is reported in this publication. For details of the other reported outcomes, please see Supplement 4.

### Key studies identified

The only study to compare two different protein doses in malnourished oncology patients was Goodrose-Flores et al. (2020) [24], a retrospective analysis of medical records of 124 patients receiving palliative cancer care in Sweden between 2016 and 2018. The most common type of cancer was gastrointestinal (40%). One group of patients (*n* = 20) received a mean 1.15 g/kg/day of protein, while the other group (*n* = 104) received a mean 0.77 g/kg/day. Percentage weight gain from baseline was calculated after patients had received HPN for between three weeks and two months; average weight gain was significantly greater amongst patients receiving the higher of the two protein doses (3.3% vs 0.12%, *p* = 0.04). To investigate the safety of the HPN interventions, liver enzymes were assessed in 85 patients (1.15 g/kg/day group, *n* = 19; 0.77 g/kg/day group, *n* = 66), as an indicator of possible liver dysfunction. The proportion of patients with elevated liver enzymes did not differ significantly between the two treatment groups (*p* = 0.34); elevated liver enzymes were observed in 11% and 24% of patients in the 1.15 and 0.77 g/kg/day groups, respectively.

In the only RCT identified by the SLR, Obling et al. (2019) compared sHPN (sHPN and dietetic counselling) with non-sHPN (best practice nutritional care and dietetic counselling) [33]. The sHPN group did not receive significantly more energy, but protein intake was significantly higher at visits 2, 3, and 5 (95% confidence interval [CI]: 0.38, 0.47; *p* < 0.05). Overall, median protein intake ranged from 1.08–1.39 g/kg/day in the sHPN arm vs 1.10–1.16 g/kg/day in the non-sHPN arm. After 12 weeks, 69% of patients in the sHPN group had increased their fat-free mass, compared with 40% of patients in the non-sHPN group (*p* < 0.01).

The only other comparative study was the prospective observational study by Cotogni et al. (2022) [41], which compared HPN with artificial hydration. The prescribed energy of HPN was 25–30 kcal/kg/day, with prescribed protein of 1–1.5 g/kg/day, while the artificial hydration group received balanced salt solutions of 1L, 1.5L, or 2L, depending on their body mass. The results demonstrated that patients on HPN survived for significantly longer than those on artificial hydration (median overall survival was 4.3 vs 1.5 months, respectively, 95% CI: 0.015, 0.059; *p* < 0.001) [41].

Lastly, the prospective observational study by Vashi et al. (2014) [44] had a targeted protein dose of 1.5–2 g/kg/day for patients with BMI < 30 kg/m^2^, and 2–2.5 g/kg/day for patients with BMI ≥ 30 kg/m^2^. However, actual protein delivery was only 1.3–1.5 g/kg/day (within the standard range). In this publication, HPN was associated with the greatest improvements in quality of life and bodyweight at 3 months compared with baseline (global quality of life score: 54.4 vs 30.6, respectively; *p* = 0.02; weight: 65.9 vs 61.1 kg, respectively; *p* = 0.04).

## Discussion

To the best of our knowledge, this was the first SLR with the aim of comparing outcomes between malnourished oncology patients receiving low- (< 1 g/kg/day), standard- (1–‍1.5 g/kg/day), or high-protein HPN doses (> 1.5 g/kg/day). However, no studies were identified reporting on high-protein HPN in this population. Therefore, to assess the suitability of high-protein HPN in oncology patients in the absence of relevant studies identified by the SLR, a broader approach was taken and evidence from alternative settings and populations will also be discussed.

The SLR identified one study where two different protein doses were compared: Goodrose-Flores et al. (2020) [24]. This retrospective observational study examined the effect of 0.77 g/kg/day vs 1.15 g/kg/day protein HPN in malnourished oncology patients. Although the 1.15 g/kg/day group did not receive what would be considered a high-protein dose (i.e. > 1.5 g/kg/day), the results were promising and support the notion of increased protein intake. Patients in the 1.15 g/kg/day group gained significantly more weight than those in the 0.77 g/kg/day group, with no evidence that they were at greater risk of liver damage.

The same research group provide further evidence for the safety of increased protein in Schedin et al. (2020) [51]. This publication was not eligible for inclusion in the SLR, since the sample included a mixture of oncology and non-oncology patients. Schedin et al. (2020) assessed potential risk factors for catheter-related bloodstream infection (CRBSI) in palliative care patients receiving HPN, and one risk factor considered was the protein content of PN (median protein delivery was 1.20, 0.82, or 0.58 g/kg/day). This publication found no statistically significant effect of the three different protein doses of HPN on the incidence of CRBSI (*p* = 0.13). However, as both Goodrose-Flores et al. (2020) and Schedin et al. (2020) compared standard vs low rather than standard vs high-protein doses, one cannot conclude that increasing the protein dose above 1.5 g/kg/day would yield additional clinical benefits without increasing adverse events [24, 51].

The SLR identified three other studies of note, Obling et al. (2019) [33], Cotogni et al. (2022) [41], and Vashi et al. (2014) [44]. In the RCT by Obling et al. (2019), increased fat-free mass was observed in a significantly greater proportion of patients in the group receiving more protein, despite energy intakes not being significantly different. Although the protein doses were in the standard range (1–1.5 g/kg/day), this study provides further evidence supporting the use of increased protein compared with lower protein in malnourished oncology patients using HPN. In the prospective observational study by Cotogni et al. (2022) [41], patients receiving HPN survived for significantly longer than those on artificial hydration. However, it is unclear if there were differences in energy intake between the two groups, which may have implications for this result. In the prospective observational study by Vashi et al. (2014) [44], the target protein dose was 1.5–2 g/kg/day for patients with BMI < 30 kg/m^2^, and 2–2.5 g/kg/day for patients with BMI ≥ 30 kg/m^2^, making it the only publication identified by the SLR that explicitly aimed for high-protein intake. However, actual protein delivery was 1.3–1.5 g/kg/day, which potentially highlights the difficulty of meeting such high protein targets.

Bouleuc et al. (2020) reported that PN (protein range: 1.2–1.5 g/kg/day) did not improve quality of life or survival, and was associated with more serious adverse events (mainly infections) than oral feeding (*p* = 0.01) [52]. This study was not eligible for inclusion in the SLR, as it was unclear what proportion of cancer patients received PN at home as opposed to in a hospital setting. In addition, this study was not generalisable to the current research question, as in the PN arm, 46% of patients had an Eastern Cooperative Oncology Group (ECOG) performance status of 3 or 4, and therefore the study’s inclusion criteria did not comply with indications for HPN according to recent guidelines [19]. Additionally, in the PN arm, 60% of patients had gained weight or had 0–5% weight loss in the previous month, and so may not have been malnourished.

Overall, the results of the studies identified by the SLR lend support to the idea that increased protein intake could benefit malnourished oncology patients. However, since no studies evaluated high-protein doses, statistical analyses to compare the included studies were not feasible. Therefore, there is a clear need for future studies to determine optimal protein dose by comparing alternative doses within a single patient population.

In theory, the safety and efficacy of high-protein HPN is biologically plausible. Net muscle protein balance is required for increasing skeletal muscle mass, and nutrition is a potent anabolic stimulus [53]. Specifically, the postprandial increase in circulating amino acids stimulates muscle protein synthesis [53]. Winter et al. (2012) reported that in ten male patients with non-small cell lung cancer, protein synthesis was stimulated by increased amino acid provision resulting in hyperaminoacidaemia with increased peripheral glucose uptake [54]. Furthermore, administration of the branched chain amino acids leucine and valine increased skeletal muscle protein synthesis in a mouse model without any measurable effect on tumour mass [55]. Similarly, supplementation with leucine (0.052 g/kg of bodyweight) has been demonstrated to increase skeletal muscle protein synthesis in healthy elderly men [56]. Furthermore, intravenous administration of up to 2.0 g/kg/day amino acids demonstrated safety in an RCT of 474 ICU patients [57]. Taken together, these studies suggest that high-protein PN is an effective and safe practice, at least acutely. Notably, older oncology patients appear to have anabolic resistance to protein, although the same does not appear to be true for younger patients [54, 58, 59].

Research in non-PN settings and critically ill patients has demonstrated the value of increased protein intake for quality of life, prevention of sarcopenia, and mortality [60–63]. In a retrospective study of adult outpatients with advanced gastrointestinal cancer, Pimentel et al. (2021) reported that although a high-protein oral diet (2.2 ± 0.8 g/kg/day) was not associated with better muscle function as measured by handgrip strength, increased protein intake was associated with increased overall survival, compared with a low protein diet (0.8 ± 0.4 g/kg/day) [62]. Ferrie et al. (2016) [61], a double-blinded RCT of 119 critically ill patients, demonstrated that, when compared with 0.9 g/kg/day amino acids, 1.1 g/kg/day amino acids was associated with small improvements in several measures (grip strength, less fatigue, greater forearm muscle thickness, and better nitrogen balance), with no difference between groups in mortality or length of stay. In another RCT by De Azevedo et al. (2021) [63], a high-protein oral diet supplemented by PN (1.48 g/kg/day) and resistance exercise significantly improved the physical quality of life and survival of critically ill patients at 3- (*p* = 0.01) and 6-months (*p* = 0.001) compared with a control group receiving 1.19 g/kg/day. Mortality was also significantly lower (*p* = 0.006). Additionally, a recent SLR investigated the impact of protein intake on muscle mass in cancer patients; across eight included studies, protein intake < 1.2 g/kg was associated with muscle wasting, whereas protein intake > 1.4 g/kg was associated with muscle maintenance [64]. These studies were not eligible for inclusion in the current SLR, as the route of feeding was oral and/or enteral.

Two large clinical trials, EFFORT and NEXIS, should yield further valuable data regarding high-protein nutrition in the near future, although neither are perfectly aligned with the current research question. The EFFORT trial will compare protein targets of ≤ 1.2 g/kg/day and ≥ 2.2 g/kg/day, while the NEXIS trial will contrast patients on standard care with those completing an in-bed exercise regime and receiving total protein delivery of 2.0–2.5 g/kg/day. Both studies investigate ICU-based nutrition rather than HPN, and PN is not mandatory (protein targets can be met by any combination of enteral nutrition, oral supplements, and PN). Furthermore, the populations are not limited to cancer patients (critically ill patients of any kind are eligible). Similar studies should be performed with malnourished oncology patients to determine optimal HPN protein dosing, and the impact of combining nutrition and exercise to improve outcomes.

The main limitation of the present SLR was the absence of publications reporting on high-protein (> 1.5 g/kg/day) HPN in malnourished oncology patients. Although a subsequent targeted literature search identified studies demonstrating effective high-protein PN in other populations and settings, the approach has not yet been translated to HPN and oncology. Hence, in the absence of further data, the threshold between high- and low-protein content remains subjective, ensuring ongoing debate regarding optimal protein delivery. For example, while the ESPEN Expert Group [65] recommends older adults with acute or chronic illnesses consume 1.2 to 1.5 g/kg/day (and even more for those with severe illness or injury), Op den Kamp et al. 2009 [66] emphasize a baseline of 1.5 g/kg/day or 15–20% of the total caloric intake for patients with cachexia, while Bauer et al. 2019 [67] advocate for an intake ranging from 1 to 1.5 g/kg/day paired with physical exercise for patients with sarcopenia.

Furthermore, only one RCT was identified. A major limitation of non-randomised studies is when outcomes of patients with PN are compared with outcomes of patients without PN, these patients can differ. Such comparisons can be inherently biased, as patients selected for PN often present with more severe malnutrition and its associated complications than those not eligible for PN. This disparity introduces potential confounding variables, and any conclusions should be interpreted with caution.

In addition, two publications identified by the SLR involved patients from multiple European countries including the UK, but did not present results by country [35, 36]. This is notable, as the use of PN differs across Europe; in many countries PN is supplemental, whereas it is often used for intestinal failure in the UK, and thus unlikely to be supplemental.

Lastly, this SLR focused on HPN rather than PN in an inpatient setting, as the two populations are not directly comparable. Inpatients typically require short-term as opposed to long-term PN, while the incidence of CRBSIs, other complications, and mortality are also different. The SLR focused on outpatients alone because it is a more homogenous population.

## Conclusions

Despite the biological plausibility and emerging evidence from critically ill patients, at the time of writing there is a lack of evidence investigating and supporting the use of high-protein HPN in malnourished oncology patients. A minimum of 1.5 g/kg/day or > 20% of total caloric intake from protein appears to be optimal for elderly individuals and advanced cancer inpatients. However, whether this is also appropriate for HPN in oncology patients remains to be determined. Studies using a variety of designs (such as acute single arm safety studies and longer-term comparative studies with multiple protein doses) are needed to establish the efficacy and safety of this promising approach.

## Data Availability

The data that support the findings of this study are available from the corresponding author on reasonable request.

## References

[1] Pressoir M (2010). Prevalence, risk factors and clinical implications of malnutrition in French Comprehensive Cancer Centres. Br J Cancer.

[2] Hebuterne X (2014). Prevalence of malnutrition and current use of nutrition support in patients with cancer. JPEN J Parenter Enteral Nutr.

[3] Muscaritoli M (2017). Prevalence of malnutrition in patients at first medical oncology visit: The PreMiO study. Oncotarget.

[4] Caillet P (2017). Association between cachexia, chemotherapy and outcomes in older cancer patients: a systematic review. Clin Nutr.

[5] Ryan AM (2016). Cancer-associated malnutrition, cachexia and sarcopenia: the skeleton in the hospital closet 40 years later. Proc Nutr Soc.

[6] Meza-Valderrama D (2021). Sarcopenia, malnutrition, and cachexia: adapting definitions and terminology of nutritional disorders in older people with cancer. Nutrients.

[7] Arends J (2017). ESPEN expert group recommendations for action against cancer-related malnutrition. Clin Nutr.

[8] Prado CM (2009). Sarcopenia as a determinant of chemotherapy toxicity and time to tumor progression in metastatic breast cancer patients receiving capecitabine treatment. Clin Cancer Res.

[9] Prado CM (2011). Two faces of drug therapy in cancer: drug-related lean tissue loss and its adverse consequences to survival and toxicity. Curr Opin Clin Nutr Metab Care.

[10] Hua X (2019). When the loss costs too much: a systematic review and meta-analysis of sarcopenia in head and neck cancer. Front Oncol.

[11] Cardi M (2019). Prognostic factors influencing infectious complications after cytoreductive surgery and HIPEC: results from a tertiary referral center. Gastroenterol Res Pract.

[12] Chabowski M (2018). Is nutritional status associated with the level of anxiety, depression and pain in patients with lung cancer?. J Thorac Dis.

[13] Daly L (2020). The relationship between the BMI-adjusted weight loss grading system and quality of life in patients with incurable cancer. J Cachexia Sarcopenia Muscle.

[14] Planas M (2016). Prevalence of hospital malnutrition in cancer patients: a sub-analysis of the PREDyCES study. Support Care Cancer.

[15] Lodewick TM (2015). Are sarcopenia, obesity and sarcopenic obesity predictive of outcome in patients with colorectal liver metastases?. HPB.

[16] Yanni A (2019). Malnutrition in head and neck cancer patients: Impacts and indications of a prophylactic percutaneous endoscopic gastrostomy. Eur Ann Otorhinolaryngol Head Neck Dis.

[17] de Las Penas R (2019). SEOM clinical guidelines on nutrition in cancer patients (2018). Clin Transl Oncol.

[18] Bozzetti F (2009). ESPEN guidelines on parenteral nutrition: Non-surgical oncology. Clin Nutr.

[19] Arends J (2017). ESPEN guidelines on nutrition in cancer patients. Clin Nutr.

[20] Bozzetti F (2022). SINPE Position Paper on the use of home parenteral nutrition in cancer patients. Support Care Cancer.

[21] Pironi L (2020). ESPEN guideline on home parenteral nutrition. Clin Nutr.

[22] Kumpf VJ, Tillman EM (2012). Home parenteral nutrition: safe transition from hospital to home. Nutr Clin Pract.

[23] Pironi L, Regional Coordinators of SINPE (2017) Development of home artificial nutrition in Italy over a seven year period: 2005–2012. BMC Nutr 3:6

[24] Goodrose-Flores C et al (2022) High-protein compared with standard parenteral nutrition in palliative cancer care. BMJ Support Palliat Care 12(3):332–338 10.1136/bmjspcare-2019-002139 32451328

[25] Ravasco P (2019). Nutrition in cancer patients. J Clin Med.

[26] Arends J (2021). Cancer cachexia in adult patients: ESMO clinical practice guidelines☆. ESMO Open.

[27] Bauer J (2013). Evidence-based recommendations for optimal dietary protein intake in older people: a position paper from the PROT-AGE Study Group. J Am Med Dir Assoc.

[28] Heyland DK, Stapleton R, Compher C (2018). Should we prescribe more protein to critically Ill patients?. Nutrients.

[29] Hoffer LJ, Bistrian BR (2012). Appropriate protein provision in critical illness: a systematic and narrative review. Am J Clin Nutr.

[30] The Cochrane Collaboration (2011) Cochrane handbook for systematic reviews of interventions version 5.1. Higgins JPT, Green S (eds). Available from: http://handbook-5-1.cochrane.org/

[31] Centre for Reviews and Dissemination (2009) University of York. Systematic Reviews: CRDs guidance for undertaking reviews in health care. Available from: https://www.york.ac.uk/crd/guidance/

[32] Liberati A et al (2009) The PRISMA statement for reporting systematic reviews and meta-analyses of studies that evaluate healthcare interventions: explanation and elaboration. BMJ 339:b2700 10.1136/bmj.b2700 PMC2714672 19622552

[33] Obling SR (2019). Home parenteral nutrition increases fat free mass in patients with incurable gastrointestinal cancer. Results of a randomized controlled trial. Clin Nutr.

[34] Pelzer U (2010). Parenteral nutrition support for patients with pancreatic cancer. Results of a phase II study. BMC Cancer.

[35] Bozzetti F (2014). The prognosis of incurable cachectic cancer patients on home parenteral nutrition: a multi-centre observational study with prospective follow-up of 414 patients. Ann Oncol.

[36] Bozzetti F (2015). Development and validation of a nomogram to predict survival in incurable cachectic cancer patients on home parenteral nutrition. Ann Oncol.

[37] Culine S (2014). Home parenteral nutrition improves quality of life and nutritional status in patients with cancer: a French observational multicentre study. Support Care Cancer.

[38] Cotogni P (2017). Longitudinal study of quality of life in advanced cancer patients on home parenteral nutrition. Cancer Med.

[39] Cotogni P (2018). Bioelectrical impedance analysis for monitoring cancer patients receiving chemotherapy and home parenteral nutrition. BMC Cancer.

[40] Cotogni P (2020). Clinical characteristics and predictive factors of survival of 761 cancer patients on home parenteral nutrition: a prospective, cohort study. Cancer Med.

[41] Cotogni P et al (2022) Home parenteral nutrition versus artificial hydration in malnourished patients with cancer in palliative care: a prospective, cohort survival study. BMJ Support Palliat Care 12(1):114–120 10.1136/bmjspcare-2020-002343 32826263

[42] Cotogni P (2021). Comparative complication rates of 854 central venous access devices for home parenteral nutrition in cancer patients: a prospective study of over 169,000 catheter-days. JPEN J Parenter Enteral Nutr.

[43] Ma CJ (2021). Supplemental home parenteral nutrition improved nutrition status with comparable quality of life in malnourished unresectable/metastatic gastric cancer receiving salvage chemotherapy. Support Care Cancer.

[44] Vashi PG (2014). A longitudinal study investigating quality of life and nutritional outcomes in advanced cancer patients receiving home parenteral nutrition. BMC Cancer.

[45] Obling SR, Wilson BV, Kjeldsen J (2018). Home parenteral support in patients with incurable cancer. Patient characteristics of importance for catheter related complications and overall survival. Clin Nutr ESPEN.

[46] Ruggeri E (2020). Home artificial nutrition in palliative care cancer patients: impact on survival and performance status. Clin Nutr.

[47] Ruggeri E (2021). Choice of access route for artificial nutrition in cancer patients: 30 y of activity in a home palliative care setting. Nutrition.

[48] Santarpia L (2006). Predictive factors of survival in patients with peritoneal carcinomatosis on home parenteral nutrition. Nutrition.

[49] Soo I, Gramlich L (2008). Use of parenteral nutrition in patients with advanced cancer. Appl Physiol Nutr Metab.

[50] Santarpia L, Bozzetti F (2018). Acute impact of home parenteral nutrition in patients with late-stage cancer on family caregivers: preliminary data. Support Care Cancer.

[51] Schedin A et al (2020) Catheter-related bloodstream infections in palliative care patients receiving parenteral nutrition by medical home care. BMJ Support Palliat Care Sep 17:bmjspcare-2020-002331 10.1136/bmjspcare-2020-002331 32943471

[52] Bouleuc C (2020). Impact on health-related quality of life of parenteral nutrition for patients with advanced cancer cachexia: results from a randomized controlled trial. Oncologist.

[53] Prado CM, Purcell SA, Laviano A (2020). Nutrition interventions to treat low muscle mass in cancer. J Cachexia Sarcopenia Muscle.

[54] Winter A, MacAdams J, Chevalier S (2012). Normal protein anabolic response to hyperaminoacidemia in insulin-resistant patients with lung cancer cachexia. Clin Nutr.

[55] Eley HL, Russell ST, Tisdale MJ (2007). Effect of branched-chain amino acids on muscle atrophy in cancer cachexia. Biochem J.

[56] Rieu I (2006). Leucine supplementation improves muscle protein synthesis in elderly men independently of hyperaminoacidaemia. J Physiol.

[57] Doig GS (2015). Intravenous amino acid therapy for kidney function in critically ill patients: a randomized controlled trial. Intensive Care Med.

[58] Horstman AM, Sheffield-Moore M (2015). Nutritional/metabolic response in older cancer patients. Nutrition.

[59] Engelen MP, van der Meij BS, Deutz NE (2016). Protein anabolic resistance in cancer: does it really exist?. Curr Opin Clin Nutr Metab Care.

[60] Bosaeus I, Rothenberg E (2016). Nutrition and physical activity for the prevention and treatment of age-related sarcopenia. Proc Nutr Soc.

[61] Ferrie S (2016). Protein requirements in the critically Ill: a randomized controlled trial using parenteral nutrition. JPEN J Parenter Enteral Nutr.

[62] Pimentel GD (2021). High protein diet improves the overall survival in older adults with advanced gastrointestinal cancer. Clin Nutr.

[63] de Azevedo JRA (2021). High-protein intake and early exercise in adult intensive care patients: a prospective, randomized controlled trial to evaluate the impact on functional outcomes. BMC Anesthesiol.

[64] Capitao C (2022). Protein intake and muscle mass maintenance in patients with cancer types with high prevalence of sarcopenia: a systematic review. Support Care Cancer.

[65] Deutz NE (2014). Protein intake and exercise for optimal muscle function with aging: recommendations from the ESPEN Expert Group. Clin Nutr.

[66] Op den Kamp CM (2009). Muscle atrophy in cachexia: can dietary protein tip the balance?. Curr Opin Clin Nutr Metab Care.

[67] Bauer J (2019). Sarcopenia: a time for action an SCWD position paper. J Cachexia Sarcopenia Muscle.

